# The effect of randomness for dependency map on the robustness of interdependent lattices

**DOI:** 10.1063/1.4939984

**Published:** 2016-01-19

**Authors:** Jing Yuan, Lixiang Li, Haipeng Peng, Jürgen Kurths, Jinghua Xiao, Yixian Yang

**Affiliations:** 1 State Key Laboratory of Networking and Switching Technology, Beijing University of Posts and Telecommunications, Beijing 100876, China; 2 Potsdam Institute for Climate Impact Research, Potsdam D-14473, Germany; 3 School of Science, Beijing University of Posts and Telecommunications, Beijing 100876, China

## Abstract

The percolation for interdependent networks with identical dependency map follows a second-order phase transition which is exactly the same with percolation on a single network, while percolation for random dependency follows a first-order phase transition. In real networks, the dependency relations between networks are neither identical nor completely random. Thus in this paper, we study the influence of randomness for dependency maps on the robustness of interdependent lattice networks. We introduce approximate entropy(*ApEn*) as the measure of randomness of the dependency maps. We find that there is critical *ApEn_c_* below which the percolation is continuous, but for larger *ApEn*, it is a first-order transition. With the increment of *ApEn*, the *p_c_* increases until *ApEn* reaching $ApEn_{c}^{\prime }$ and then remains almost constant. The time scale of the system shows rich properties as *ApEn* increases. Our results uncover that randomness is one of the important factors that lead to cascading failures of spatially interdependent networks.

The interdependent networks which fully consider the interactions between networks have been used to model real complex systems better. Robustness is one of the most important properties for interdependent networks especially spatially interdependent networks, since most of the infrastructure networks are spatially networks. In real interdependent networks, the dependency relationship is not usually random. Thus, we analyze how the randomness of dependency map affects the robustness of interdependent lattices which are used to model the spatially interdependent networks. We found that the randomness of dependency map between networks is quite critical for the robustness of interdependent lattices.

## I. INTRODUCTION

Robustness is one of the most important properties of complex networks and has been widely explored on single networks in the last decade.^1–8^ However, complex systems are rarely isolated. The more casual situation is that networks usually interact with other networks such as transportation networks and financial systems.^9–13^ In the case of interdependent networks, conclusions are often far different from single networks. In particular, a removal of a very small fraction of nodes can lead to catastrophic failures on the whole network.^14^ A theoretical framework based on percolation theory has been established to analyze the resilience of interdependent systems,^9,15^ and much details have been explored.^16–20^ The fraction of interdependent nodes is a key factor that will influence the phase transition of the networks.^21,22^ Also, the overlap of links can significantly change the properties of the percolation, and there is a critical point above which the emergence of the mutually connected component is continuous.^20^ The presence of degree correlations in multiplex networks can modify drastically the percolation threshold.^18,19^

Most previous models have focused on interdependent random and scale-free networks in which space restrictions are not considered. However, many real-world systems such as power grid networks and computer networks are embedded in two-dimensional space.^23,24^ In interdependent random and scale-free networks, the overlap of links and degree correlations will change the properties of phase transition. Nevertheless, for spatially embedded interdependent networks which are modeled as square lattices, the overlap of links or the degree correlations of nodes lose significance, since their network topologies are identical. The spatially interdependent networks are extremely vulnerable. Any fraction of interdependent nodes will lead to first-order transition.^23^ From an identical dependency map to totally random dependency map, the randomness of the dependency map may be one of the most important factors leading to the emergence of discontinuous percolation. In most real interdependent systems, dependencies are neither totally random nor identical. Researches on the resilience of intermediate systems that lie somewhere between these two extremes are of high practical significance and need further exploration.

From this perspective, we study the relationship between the dependency's randomness and stability of the system of two interdependent spatially embedded networks. We use approximate entropy(*ApEn*) as the measure of randomness. One of the big challenges here is how to introduce controlled degree of randomness into the system. Therefore, we propose an intermediate model which describes the system with dependency map between identical map and totally random map. Inspired by the constructing procedure of the Watt-Strogatz small-world model,^25^ starting from an identical dependency map, we rewire each dependency link at random with probability *q*. By increasing *q* from 0 to 1, the *ApEn* increases monotonically. Therefore, the traverse of randomness can be generally represented by *q*. We reveal that there is a critical value *q_c_*, below which the percolation transition becomes continuous, whereas for any *q* > *q_c_*, the collapse is discontinuous. Changing the topologies on a single layer, we discover that *q_c_* is different for interdependent scale-free networks, Watts-Strogatz networks, and Erdős-Rényi networks. There is another critical value $q\prime_{c}$ for the function *p_c_* VS *q*, which is different from *q_c_*. The percolation threshold *p_c_* increases with *q* when $q<q_{c}^{\prime }$ and remains approximately constant when $q>q_{c}^{\prime }$. Additionally, we present an analytical method for time scale of cascade failures based on critical p and find that the four topologies display rich transient properties when *q* changes from 0 to 1. Finally, we analyze the influence of limited dependency length on spatial networks. With the same dependency length, we show that a linearly dependent system is always continuous, but not continuous for some locally randomly dependent system. Our results show that the randomness of dependency may be one of important factors for extreme vulnerability of spatially interdependent systems.

## II. MODEL DESCRIPTION

Our model of interdependent networks is realized via two networks (*N* = 10^6^) A and B under full dependency. Here, one network is the copy of the opposite network and their average degree ⟨*k*⟩ = 4 (the same as a square lattice). The degree distribution of the scale-free network is ⟨*k*⟩^−*λ*^, where *λ* = 2.7. In each network, each node has two types of links: connectivity link and dependency link. Also, every node in network A is connected with one and only one node in network B. For a square lattice, each node is connected to its four nearest neighbors within the same lattice via connectivity links. All dependencies in our model are mutual and bidirectional. Dependency is taken to mean that if a node in network A is removed from the system and a node in B that depends on it will be removed from B as well. Thus failures of nodes iterate until mutually connected giant component of both networks emerges. This process is called cascade failures and see Methods for details of cascade process of the system.

**FIG. 1.**
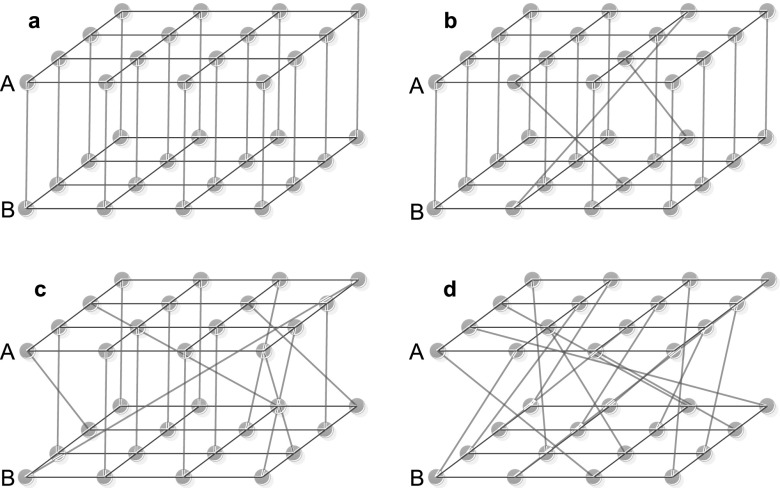
The interdependent square lattices with the rewiring probability of dependency links *q* = 0, 0.25, 0.50, 1.00, respectively. When *q* = 0, the dependency map is an identical mapping, i.e., node *i* in network A is dependent on node *j* in network B, where *i* = *j* (Fig. 1(a)). When *q* = 1.00, the situation is the same as totally random mapping (Fig. 1(d)). When *q* = 0.25 *or* 0.5, the situation is between both extremes (Figs. 1(b) and 1(c)).

There are two extreme situations. (i) node *i* in A depends on node *j* in the B such that *j* = *i*. We call it identical dependency map (Fig. 1(a)). (ii) The random dependency map as most papers considered (Fig. 1(d)). Like the constructing procedure of the Watt-Strogatz small-world model, starting from the identity dependency map, we rewire each dependency link at random with probability *q*, while guaranteeing that each node in A depends on one and only one node in B(0 ≤ *q* ≤ 1). We sample *q* = 0, 0.25, 0.50, 1 and plot them in Fig. 1.

**FIG. 2.**
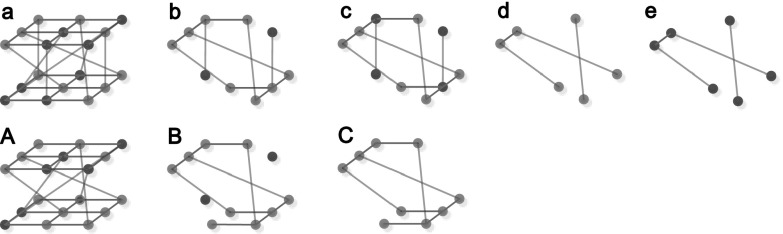
Difference of cascade failures between partially randomly interdependent lattices and partially interdependent lattices. The blue points stand for the survived nodes, while the red points stand for the failure nodes. Figure sequence (a)–(e) stands for the cascade failures process in partially randomly interdependent lattices with *q* = 5/9 (fraction of nodes that are randomly dependent and the remaining 1 − *q* of nodes are dependent with the identical nodes in the opposite network) and *p* = 4/9 (fraction of nodes initially removed). Figure sequence (A)–(C) stands for the cascade failures process in partially interdependent networks with *q* = 5/9 (fraction of nodes that are dependent and the remaining 1 − *q* are autonomous) and *p* = 4/9. It can be obviously seen with the same *q*, the size of giant component in partially randomly interdependent lattices is 0/9, while the size of giant component in partially interdependent lattices is 4/9. The cascade failures process differs for these two models.

Note: We must figure out that our model is different from partially interdependent lattices proposed by Bashan *et al*.^23^ In partially interdependent lattices, there are interdependent lattices with a fraction *q* of interdependent nodes and the remaining 1 − *q* of nodes autonomous. In our model, however, the remaining 1 − *q* nodes are connected with the identical nodes in the opposite network. It is illustrated in Fig. 2. In Fig. 2, we can see that the cascade failures process differs much between these two models: with the same *q* = 5/9 and *p* = 4/9, the size of the giant component in our model is 0/9, while the size of giant component in partially interdependent networks is 4/9. This apparently shows that our model is different from partially interdependent lattices.

## III. RESULTS

**FIG. 3.**
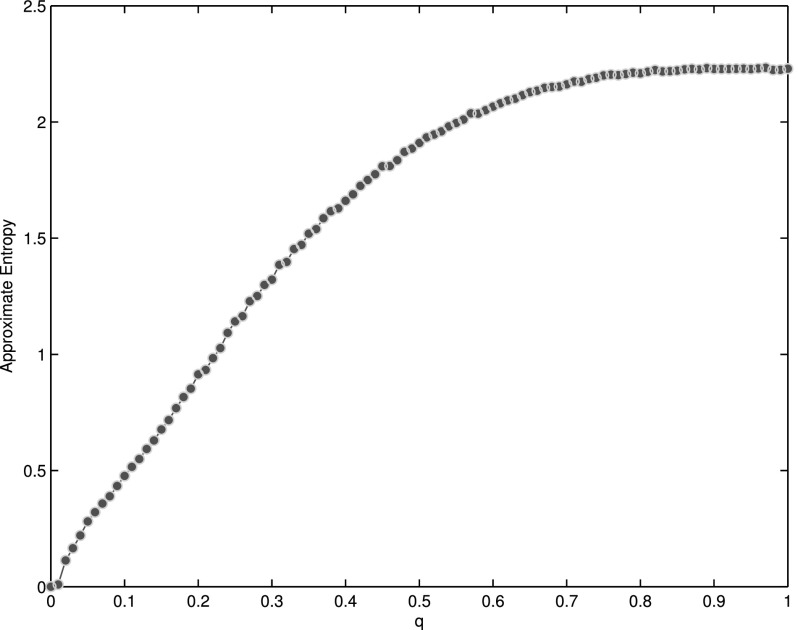
The value of *ApEn* under different *q*. When *q* = 0, *ApEn* is nearly 0, and when *q* = 1, *ApEn* reaches its maximum. The *ApEn* is a continuous function of *q*, and it changes monotonously as the increment of *q*.

Entropy can be used to measure the randomness effectively.^26^ In fact, approximate entropy(*ApEn*) is adopted in this paper for computation convenience. When *q* = 0, *ApEn* is nearly 0, and when *q* = 1, it reaches its maximum. The *ApEn* is a continuous and monotonically increasing function of *q* (Fig. 3). However, the randomness is not fully represented by rewiring dependency links, since the locally randomly interdependent lattices^27^ in which *q* = 1 is not totally randomly interdependent but with length constraints. Then, considering a more casual situation, the permutation of 1–*N* cannot be exhausted by rewiring the dependency links of identical map at probability *q*. But as the approximate entropy changes continuously with *q*, we can traverse *q* to generally represent all approximate entropies.

**FIG. 4.**
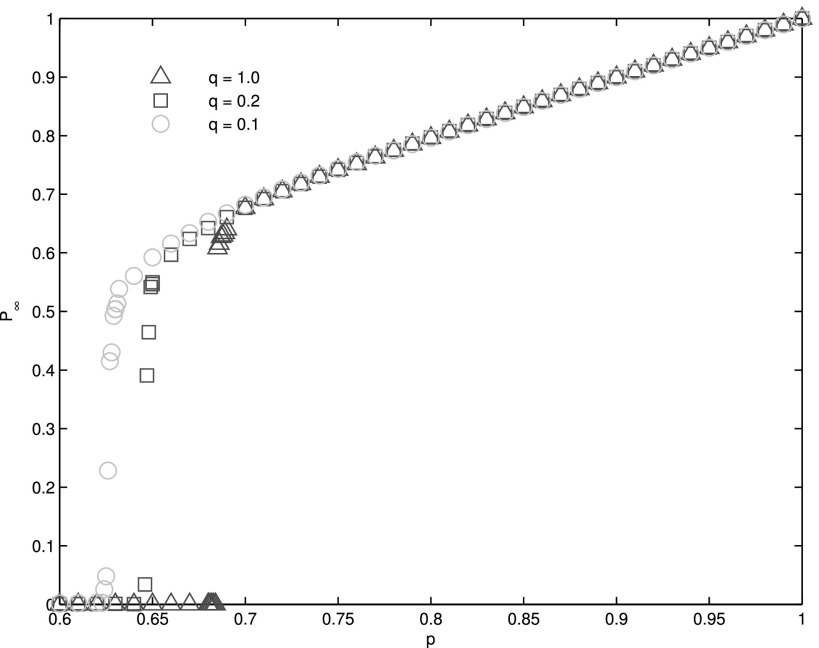
Relations of the size of *p_∞_* at steady state after random failure of a fraction 1 − *p* (Δ*p* = 10^−3^) of the nodes on two interdependent square lattices, each of size 1000 × 1000. The green circles, red squares, and blue triangles stand for *q* = 0.1, *q* = 0.2, and *q* = 1.0, respectively. The numerical results are obtained by averaging 100 realizations of networks. For *q* = 0.1, the phase transition of the system is second-order since the giant component emerges in multiple size steps (Δ*p*). For *q* = 0.2 and *q* = 1.0, however, the transition is first-order as the giant component collapses even by removing a very small fraction of nodes.

**FIG. 5.**
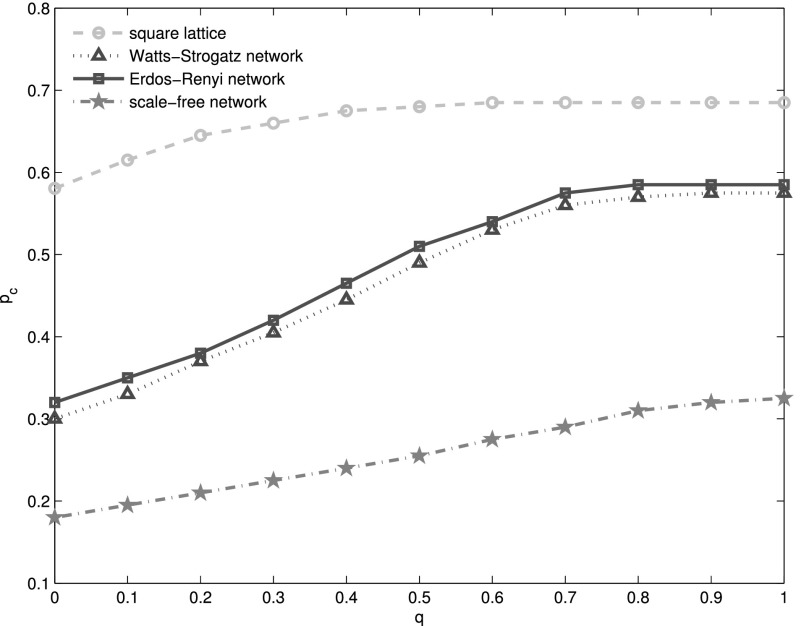
Percolation threshold *p_c_* VS *q*. The cyan circles, blue triangles, red squares, and brown stars stand for *p_c_* of interdependent square lattices, Watt-Strogatz networks, Erdös-Rényi networks, and scale-free networks, respectively. There is a critical $q\prime_{c}$, below which the *p_c_* increases almost linearly with *q*, while *p_c_* remains almost constant when $q\geq q\prime_{c}$. *p_c_* for interdependent square lattices is greater than other three networks. This means that interdependent square lattices are the most vulnerable for random attacks, while the interdependent scale-free networks are the most stable system. For scale-free networks, *λ* = 2.7. And in Watts-Strogatz networks, the rewiring probability equals 0.5. The average degree of each network in all the four systems, i.e., ⟨*k*⟩ = 4. The numerical results are obtained by averaging 100 realizations of networks consisting of *N* = 10^6^ nodes.

Through simulation, we find that there is a critical *q_c_* ≈ 0.13 for a system of interdependent lattice networks below which the percolation is second-order but first-order above. In Fig. 4, we can see that for *q* = 0.1, the phase transition of the system is second-order since the decrease of giant component occurs in multiple size steps (Δ*p*). For *q* = 0.2 and *q* = 1.0, the giant component may completely collapse by removal of a small fraction of nodes, characteristic of a first-order transition (Fig. 4). There is another critical value $q\prime_{c}$, which is different from *q_c_*. The variation tendency of the percolation threshold *p_c_* on the left side of $q\prime_{c}$ is distinct from right side of it. When *q* is relatively small, *p_c_* increases approximately linearly with *q*. And when $q>q\prime_{c}$, *p_c_* remains almost constant (Fig. 5). In other words, when $q<q\prime_{c}$, the more random the dependency map is, the more fragile the system is.

**FIG. 6.**
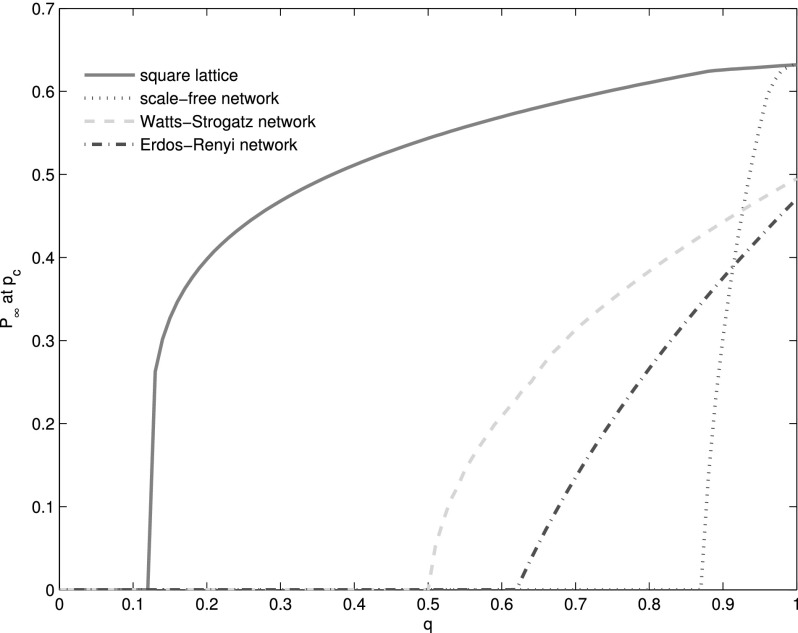
Size of giant component at *p_c_* VS *q*. The brown, blue, cyan, and red lines stand for size of giant component at *p_c_* of interdependent square lattices, scale-free networks, Watt-Strogatz networks, and Erdős-Rényi networks, respectively. It can be clearly observed that $q_{c}^{sl}<q_{c}^{WS}<q_{c}^{ER}<q_{c}^{SF}$. For scale-free networks, *λ* = 2.7. And in Watts-Strogatz networks, the rewiring probability equals 0.5. The average degree of each network in all the four systems, i.e., ⟨*k*⟩ = 4. The numerical results are obtained by averaging 100 realizations of networks consisting of *N* = 10^6^ nodes.

Analogously, for interdependent scale-free networks, Watts-Strogatz networks, and Erdős-Rényi networks, there is also a critical *q_c_*. As the critical *q_c_* is different for different interdependent networks, we define the $q_{c}^{sl}$ as *q_c_* of interdependent square lattices. Similarly, we use $q_{c}^{WS},\;q_{c}^{ER}$, and $q_{c}^{SF}$ to stand for *q_c_* of interdependent Watts-Strogatz networks, Erdős-Rényi networks, and scale-free networks, respectively. We find that $q_{c}^{sl}=0.13<q_{c}^{WS}=0.52<q_{c}^{ER}=0.61<q_{c}^{SF}=0.87$ (Fig. 6). Additionally, *p_c_* of lattice network is generally greater than that of other networks. This means that a system of interdependent scale-free networks is most robust under random attacks, while the system of interdependent square lattices is most vulnerable. A system of interdependent random networks is more stable than a small-world one (Fig. 5).

**FIG. 7.**
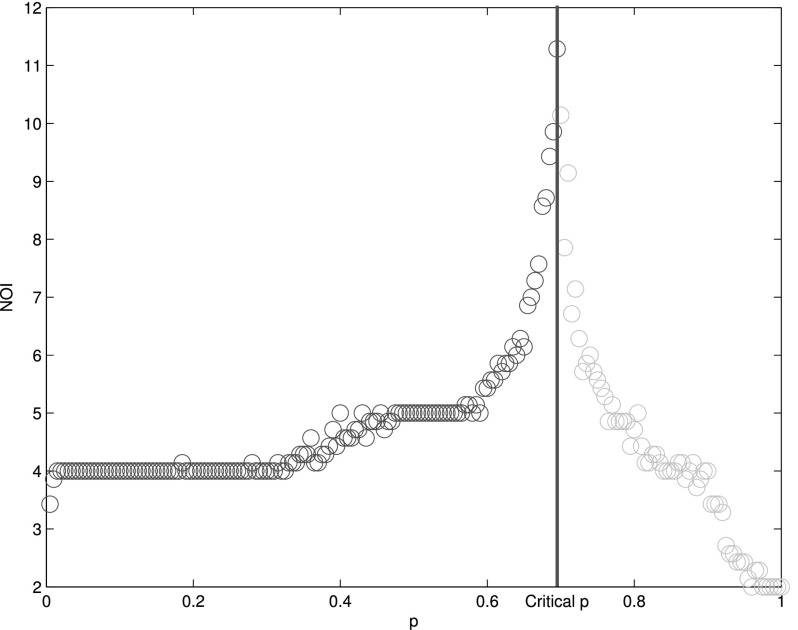
The function of number of iterations (NOI) VS *p* (Δ*p* = 10^−2^) in interdependent lattice networks (*q* = 1). The numerical results are obtained by averaging 100 realizations of networks consisting of *N* = 10^4^ nodes. The vertical red line is the critical line. On the left side of it, the system collapses down (blue circle), while a giant component remains functional on the right side (green circle). There is a sharp divergence of the NOI when *p* approaches *p_c_*.

**FIG. 8.**
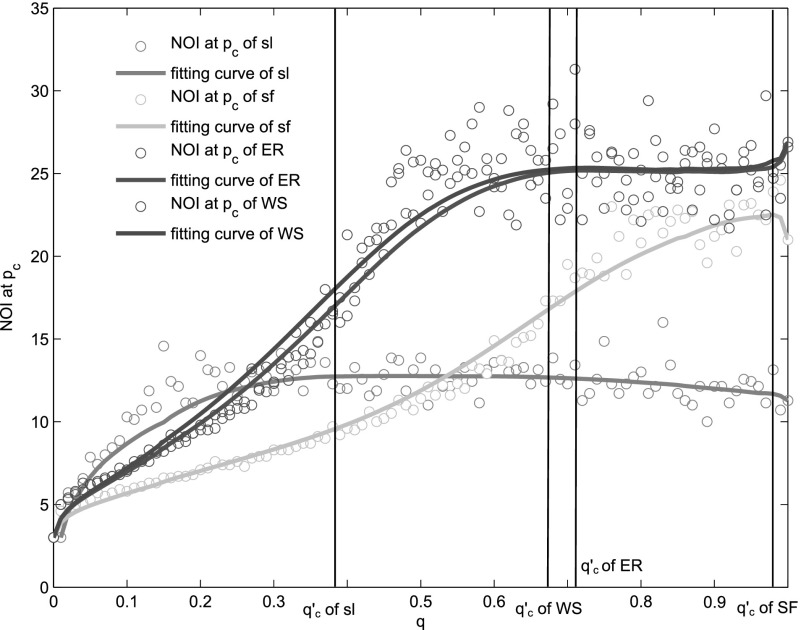
The change of NOI at *p_c_* with *q*. The brown, cyan, red, and blue circles stand for NOI at *p_c_* of interdependent square lattices, scale-free networks, Watt-Strogatz networks, and Erdös-Rényi networks, respectively. For interdependent random networks and small-world networks, NOI increases monotonously with *q*. However, for interdependent lattice networks and scale-free networks, there is one critical $q\prime_{c}$. When $q<q\prime_{c}$, the NOI increases approximately linearly, and when $q\geq q\prime_{c}$, the *NOI* starts to decreases. For scale-free networks, *λ* = 2.7. And in Watts-Strogatz networks, the rewiring probability equals 0.5. The average degree of each network in all the four systems, i.e., ⟨*k*⟩ = 4. The numerical results are obtained by averaging 100 realizations of networks consisting of *N* = 10^4^ nodes.

The time scale of cascade failures, i.e., the time that the interdependent networks needed to collapse to the stationary state is an evidently important merit for system's resilience. When the system's phase transition is first-order, the number of iterations (*NOI*) increases and reaches its peak at *p_c_* and goes quickly down to a small value with *p* (Fig. 7). And when the system's phase transition is second-order, the number of iterations varies little with *q*. So, the *NOI* at *p_c_* is an effective measure for time scale of the system. The *NOI* at *p_c_* (i.e., $NOI_{p_{c}}(q)$) is a function of *q*. $NOI_{p_{c}}(q)$ increases quickly with *q* when $q<q\prime_{c}$ and declines very gently above $q\prime_{c}$ for interdependent lattice networks. For interdependent scale-free network, it increases until $q\prime_{c}$ and then starts to decline. For interdependent random networks and small-world networks, it increases monotonously with *q*, but the variation tendency becomes nearly 0 above $q\prime_{c}$ (Fig. 8). All four interdependent systems have variation tendency's changes around their own $q\prime_{c}$. $NOI_{p_{c}}(q)$ of interdependent lattice networks is greater than those of scale-free, small-world, and random networks when $q<q_{c}^{sl}$. $NOI_{p_{c}}(q)$ of interdependent square lattice is smaller than those of all other three network types when $q>q_{c}^{ER}$. The NOI reflects the time scale of system collapse. Our results show that the transient characteristics of the four systems go through rich changes with the variation of *q*.

On the other hand, *NOI* strongly depends on system size. Therefore, take interdependent square lattices as example, we get simulation data of $NOI_{p_{c}}(q)$ from systems whose size ranges from *N* = 10^2^ to *N* = 10^6^. It is also true that $NOI_{p_{c}}(q)$ increases quickly with *q* when $q<q\prime_{c}$ and then declines very gently when *q* is greater than $q\prime_{c}$ for interdependent lattice networks. However, the critical value $q\prime_{c}$ converges gradually from a relatively great value to around 0.38 with the increment of system size. The relationship between critical value $q\prime_{c}$ and system size is shown in Table I.

**FIG. 9.**
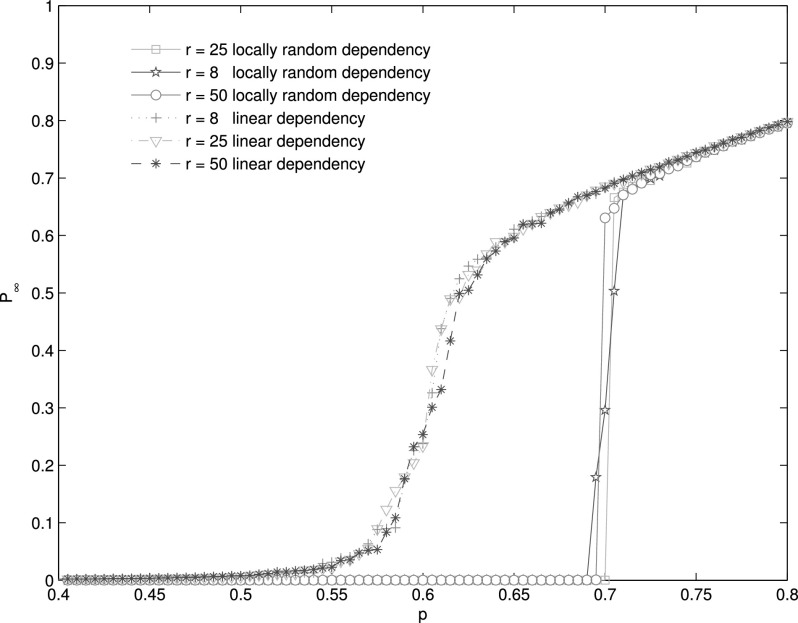
The fraction of nodes in the giant component as a function of *q*. For locally random interdependent network, when *r* = 8, the system represents the characteristic of a second-order transition. For *r* = 25 and *r* = 50, the giant component may completely collapse by removal of even a single additional node, which represents the characteristic of a first-order transition. However, for linearly interdependent networks, the transitions are second-order one when *r* = 8, 25, 50, and even *r* = *L*. The numerical results are obtained by averaging 100 realizations of networks consisting of *N* = 10^6^ nodes.

Finally, we check locally interdependent networks in which the distance between two interdependent nodes is limited (*d* ≤ *r*, i.e., $|x_{1}-x_{2}|\leq r$ and $y_{1}-y_{2}|\leq r$ in Reference 27). For simplicity, we consider one more special condition. Here, we split the whole network into small blocks of size *r* * *r*, and dependency links are randomized within each block. We find that there is critical distance *r_c_* ≈ 25 under which the percolation is continuous but discontinuous above *r_c_* (Fig. 9). The *r_c_* is greater than $r_{c}^{\prime }$ in Ref. 27 because the randomness(approximate entropy) here is lower than that in Ref. 27 with the same distance. The corresponding approximate entropy *ApEn* ≈ 0.923 of *r_c_* is approximately equal to *ApEn_c_*. Compared with locally random dependency, the linear dependency map is more robust. In linear dependency map in this paper, each node (*i*, *j*) in network of size *L* × *L* is mapped to node (i+c mod L,j+b mod L), where c and d are integers. In fact, it is also an isometric mapping. In linear dependency map, the distance of dependency link *r* = *max*(*c*, *d*). For the linear dependency map, the percolation is always continuous (Fig. 9). Although the dependency distance changes strongly, the approximate entropies of those dependency maps are almost equal to 0. So, their percolation properties are nearly the same as percolation on a single lattice. It is thus clear that the randomness may be a more important factor leading to cascade failures than dependency distance.

Furthermore, it is possible that the randomness of dependency is related to other metrics of interdependent networks such as dimension. The dimension of networks is a function of the distribution of link lengths.^28^ For spatially embedded networks, the dimension is one of the most fundamental quantities to characterize its structure and very likely will influence its percolation property.^28^ However, to the best of our knowledge, how interdependency relationships between networks change the dimension of the system has not been figured out so far. In Reference 27, the authors discovered that the dependency length plays a critical role in the percolation transition. However, we find that under linear dependency map, the change of dependency length influences the percolation property little. From the discrepancy of those two situations, it can be inferred that local property of dependency relationship makes a notable difference. And, the local property of dependency will directly influence the local topological inter-similarity between networks. Randomness happens to reflect the local property of dependency (we can see this from the computation steps of approximate entropy in Section Methods). In spatially interdependent embedded networks, the local characteristics of dependency can be more intuitively characterized as the relative length of dependency links. Under linear dependency map, the relative length of dependency links and the approximate entropy of dependency map are nearly 0. No matter how large *r* is, they change little and remain nearly 0, so the percolation changes little. On the other hand, the smaller the relative length is, the less dimension is changed from that of single network. There should exist some relations between dimension and the randomness of the dependency map.

## IV. DISCUSSION

In many real interdependent systems such as coupled power grid and communication network, the dependency relationship is neither completely regular nor completely random but lies somewhere between these two extremes. The transition from regular to random dependency is one of the keys to extreme vulnerability of spatially interdependent systems. From the proposed intermediate cascade failure model (from regular to random dependency), we find there is a transform from continuous percolation to discontinuous percolation with the randomness variation of the dependency map between two interdependent networks. We emphasize the generic character of our model because the dependency map could influence not only the resilience but also synchronization, disease spreading, and other dynamic processes in interdependent networks. With suitable modification, our model could be applied to understand the dynamical process in most real interdependent systems since the dependency maps between networks are more various and complicated instead of totally random dependency or regular dependency.

The time scale of cascade failures is essential for system's resilience, but it has received little attentions in the analysis of resilience so far. In different dynamic processes, the characteristic time scales of systems vary greatly. For instance, biological systems, social systems, and financial market dynamics have time scale much longer than that of cascade failures of power grid. Our analytic method based on critical p is simple and effective for characterizing the time scale of different systems. Generally, the system which has a shorter time scale demands higher requirements for our responding speed to catastrophe and brings much bigger challenges for us to take mitigation actions than those with longer time scale. Therefore, our method may provide a clue for research on revealing the transient mechanism and mitigation of cascade failures in interdependent networks.

## V. METHODS

### A. Approximate entropy

The randomness for the dependency maps of the interdependent square lattices is measured by approximate entropy. Entropy can effectively reflect the randomness of a sequence. However, for computation convenience, we choose the approximate entropy as the measure of randomness for the dependency maps. The approximate entropy is denoted by *ApEn* and is computed by following steps:^26,29^

(A) Given a series
  X(i)=[u(i),u(i+1),…,u(i+m−1)], i=1∼N−m+1.
(B) Count the distance between the vector *X*(*i*) and other *X*(*j*) for each i
  $$d[X(i),X(j)]=\underset{k=0∼m-1}{\max}|u(i+k)-u(j+k)|.$$
(C) Given an threshold, count the ratio between the number such that *d*[*X*(*i*), *X*(*j*)] < *r* for each i and the number of the vectors, i.e., *N* − *m* + 1(denoted by ${\;}_{i}^{m}(r)$). as
  $$C_{r}^{m}(r)=\frac{\left\{the\;number\;of(d[X(i),X(j)])<r\right\}}{N-m+1}.$$
  Generally, $C_{r}^{m}(r)$ reflects m-dimensional pattern
(D) $\phi^{m}(r)=(\frac{1}{N-m+1})\sum_{i=1}^{N-m+1}lnC_{i}^{m}(r)$
(E) $ApEn(m,r)=\Phi^{m}(r)-\Phi^{m+1}(r)$.

Parameter selection: here, we choose *m* = 2 and *r* = 0.2* (*standard deviation of u*).

### B. Percolation transition

The percolation transition is studied by randomly removing a fraction 1 − *p* of nodes and the links attached to them from both networks simultaneously. Then, on each network, clusters which are detached from the largest connected component are removed. After that, the nodes in each network which lost their supporting nodes in the opposite network are removed. This, in turn, causes more clusters to break off from the giant component, and this process is continued until no more clusters break off. First, we analyze the situation with totally random dependency maps. After the initial attack, only a fraction *p*_1_ = *p_∞_*(*p*) of nodes remains functional. Each node in A that is removed causes the removal of its interdependent node in B. Then only *p_∞_*(*p*_1_) nodes in B remain alive. This produces further damage in A and causes cascading failures. The cascade failures can be represented by the recursive equation for the survived fraction *p_i_*^14,27^

$$\begin{array}{c} p_{0}=p,\\ p_{1}=\frac{p}{P_{0}}p_{\infty }(p_{0})=P_{\infty }(p),\\ \cdots \\ p_{i}=\frac{p}{P_{i-1}}p_{\infty }(p_{i-1}). \end{array}$$ (1)

In the limit *i* → *∞*, Eq. (1) yields the equation for the mutual giant component at the steady state

$$x=\sqrt{pP_{\infty }(x)}.$$ (2)

Equation (2) can be solved graphically by the intersection between the curve *y* = *pP_∞_*(*x*) and the straight line *y* = *x*. Next, we consider the mutual percolation for more casual situations where the dependency is not totally random. For every dependency link, there is a probability *q* to rewire it at random. This is equivalent to the situation with a fraction 1 − *q* of nodes mapping to itself and the remaining *q* nodes having a random dependency map. The case of *q* = 1 corresponds to the scenario of a random dependency map, and *q* = 0 is identical to the conventional percolation on a single lattice. For the initial attack which destroys a fraction 1 − *p* of nodes, ⌊m=(1−p)N⌋ nodes are removed. We compute the probability *P_same_* that one node in A depends on the same node in B. For *n* nodes in totally random dependency networks, the number of nodes *E*(*n*) in the same location of both networks is^30^

$$E(n)=\frac{\sum_{m=0}^{n}m*C_{n}^{m}D(n-m)}{n!},$$ (3)

$$D(n)=n!\sum_{k=2}^{n}{\left(-1\right)}^{k}*n!/k!.$$ (4)

When *n* is very large, the computation of *D*(*n*) is very inconvenient. For computation simplification, when *n* ≥ 2, we have

D(n)≈⌊n!/e+0.5⌋, (5)

where *e* is the Euler's number and ⌊x⌋ is the integer part of *x*. Then

$$\begin{array}{c} E(n)=\frac{\sum_{m=1}^{n}\frac{m*n!}{(n-m)!m!}\left\lfloor \left(n-m)!/e+0.5\right)\right\rfloor }{n!},\\ \approx \frac{\sum_{m=1}^{n}\frac{m*n!}{(n-m)!m!}(n-m)!/e+0.5)}{n!},\\ =\sum_{m=1}^{n}\frac{1}{e(m-1)!}+\frac{1}{2(n-m)!(m-1)!},\\ =1+\sum_{m=0}^{n}\frac{1}{2(n-m+1)!m!}\leq 1+\frac{e}{2}. \end{array}$$ (6)

So for each node, the probability that it is in the same location of A and B is

$$P_{same}=(1-q)*p+p*\frac{E(q*N)}{q*N}.$$ (7)

When *n* → *∞*, *P_same_* → (1 − *q*) * *p*. The initial attack causes some number of nodes to be disconnected from the giant component in both networks A and B. Furthermore, because of the dependency relationship, the nodes disconnected from A will lead to further damages. *P_∞_* increases with *P_same_*. The greater the *P_same_* is, the more nodes disconnected from the giant component of A overlap the nodes in B. So, further damage decreases and cascade failures are weakened (or prevented) from the beginning. For *q* = 0, the cascade failures are prevented from the beginning and the percolation is continuous. When *q* = 1, the totally random dependency map will lead to a first-order transition. As *q* increases, the percolation transition changes from a continuous transition to a discontinuous one. There must be a critical *q_c_* beyond which the percolation transition becomes discontinuous.

## Figures and Tables

**TABLE I.** The critical values of $NOI_{p_{c}}(q)$ VS system size.

System size	10^2^	1.6 × 10^3^	10^4^	1.6 × 10^5^	10^6^
Critical value	0.61	0.43	0.40	0.39	0.38

## References

[1] M. E. Newman , “ The structure and function of complex networks,” SIAM Rev. 45, 167–256 (2003). 10.1137/S003614450342480

[2] D. S. Callaway , M. E. Newman , S. H. Strogatz , and D. J. Watts , “ Network robustness and fragility: Percolation on random graphs,” Phys. Rev. Lett. 85, 5468 (2000). 10.1103/PhysRevLett.85.5468 11136023

[3] R. Cohen , K. Erez , D. Ben-Avraham , and S. Havlin , “ Resilience of the internet to random breakdowns,” Phys. Rev. Lett. 85, 4626 (2000). 10.1103/PhysRevLett.85.4626 11082612

[4] R. Cohen , K. Erez , D. Ben-Avraham , and S. Havlin , “ Breakdown of the internet under intentional attack,” Phys. Rev. Lett. 86, 3682 (2001). 10.1103/PhysRevLett.86.3682 11328053

[5] R. Cohen , D. Ben-Avraham , and S. Havlin , “ Percolation critical exponents in scale-free networks,” Phys. Rev. E 66, 036113 (2002). 10.1103/PhysRevE.66.036113 12366190

[6] R. Albert , H. Jeong , and A.-L. Barabási , “ Error and attack tolerance of complex networks,” Nature 406, 378–382 (2000). 10.1038/35019019 10935628

[7] A. Vázquez and Y. Moreno , “ Resilience to damage of graphs with degree correlations,” Phys. Rev. E 67, 015101 (2003). 10.1103/PhysRevE.67.015101 12636544

[8] P. Crucitti , V. Latora , M. Marchiori , and A. Rapisarda , “ Efficiency of scale-free networks: Error and attack tolerance,” Physica A 320, 622–642 (2003). 10.1016/S0378-4371(02)01545-5

[9] J. Gao , S. V. Buldyrev , H. E. Stanley , and S. Havlin , “ Networks formed from interdependent networks,” Nat. Phys. 8, 40–48 (2012). 10.1038/nphys2180 23005189

[10] A. Cardillo , M. Zanin , J. Gómez-Gardeñes , M. Romance , A. J. G. del Amo , and S. Boccaletti , “ Modeling the multi-layer nature of the European air transport network: Resilience and passengers re-scheduling under random failures,” The Eur. Phys. J. Spec. Top. 215, 23–33 (2013). 10.1140/epjst/e2013-01712-8

[11] R. Criado , B. Hernandez-Bermejo , and M. Romance , “ Efficiency, vulnerability and cost: An overview with applications to subway networks worldwide,” Int. J. Bifurcation Chaos 17, 2289–2301 (2007). 10.1142/S0218127407018397

[12] R. Criado , M. Romance , and M. Vela-Pérez , “ Hyperstructures, a new approach to complex systems,” Int. J. Bifurcation Chaos 20, 877–883 (2010). 10.1142/S0218127410026162

[13] A. Cardillo , J. Gómez-Gardeñes , M. Zanin , M. Romance , D. Papo , F. del Pozo , and S. Boccaletti , “ Emergence of network features from multiplexity,” Sci. Rep. 3, 1344 (2013). 10.1038/srep01344 23446838 PMC3583169

[14] S. V. Buldyrev , R. Parshani , G. Paul , H. E. Stanley , and S. Havlin , “ Catastrophic cascade of failures in interdependent networks,” Nature 464, 1025–1028 (2010). 10.1038/nature08932 20393559

[15] M. M. Danziger , A. Bashan , Y. Berezin , L. M. Shekhtman , and S. Havlin , “ An introduction to interdependent networks,” Nonl. Dyna. Elec. Sys. ( Springer, 2014), pp. 189–202.

[16] S. Boccaletti , G. Bianconi , R. Criado , C. Del Genio , J. Gómez-Gardeñes , M. Romance , I. Sendina-Nadal , Z. Wang , and M. Zanin , “ The structure and dynamics of multilayer networks,” Phys. Rep. 544, 1–122 (2014). 10.1016/j.physrep.2014.07.001 32834429 PMC7332224

[17] Y. Berezin , A. Bashan , M. M. Danziger , D. Li , and S. Havlin , “ Localized attacks on spatially embedded networks with dependencies,” Sci. Rep. 5, 8934 (2015). 10.1038/srep08934 25757572 PMC4355725

[18] S. V. Buldyrev , N. W. Shere , and G. A. Cwilich , “ Interdependent networks with identical degrees of mutually dependent nodes,” Phys. Rev. E 83, 016112 (2011). 10.1103/PhysRevE.83.016112 21405749

[19] R. Parshani , C. Rozenblat , D. Ietri , C. Ducruet , and S. Havlin , “ Inter-similarity between coupled networks,” Eur. Phys. Lett. 92, 68002 (2010). 10.1209/0295-5075/92/68002

[20] D. Cellai , E. López , J. Zhou , J. P. Gleeson , and G. Bianconi , “ Percolation in multiplex networks with overlap,” Phys. Rev. E 88, 052811 (2013). 10.1103/PhysRevE.88.052811 24329322

[21] D. Zhou , J. Gao , H. E. Stanley , and S. Havlin , “ Percolation of partially interdependent scale-free networks,” Phys. Rev. E 87, 052812 (2013). 10.1103/PhysRevE.87.052812 23767589

[22] R. Parshani , S. V. Buldyrev , and S. Havlin , “ Critical effect of dependency groups on the function of networks,” Proc. Natl. Acad. Sci. U.S.A. 108, 1007–1010 (2011). 10.1073/pnas.1008404108 21191103 PMC3024657

[23] A. Bashan , Y. Berezin , S. V. Buldyrev , and S. Havlin , “ The extreme vulnerability of interdependent spatially embedded networks,” Nat. Phys. 9, 667–672 (2013). 10.1038/nphys2727

[24] M. M. Danziger , A. Bashan , Y. Berezin , and S. Havlin , “ Interdependent spatially embedded networks: Dynamics at percolation threshold,” in 2013 International Conference on Signal-Image Technology and Internet-Based Systems (SITIS) ( IEEE, 2013) pp. 619–625.

[25] D. J. Watts and S. H. Strogatz , “ Collective dynamics of ‘small-world’ networks,” Nature 393, 440–442 (1998). 10.1038/30918 9623998

[26] S. M. Pincus and A. L. Goldberger , “ Physiological time-series analysis: what does regularity quantify?” Am. J. Phys. 266, H1643 (1994). 10.1152/ajpheart.1994.266.4.H1643 8184944

[27] W. Li , A. Bashan , S. V. Buldyrev , H. E. Stanley , and S. Havlin , “ Cascading failures in interdependent lattice networks: The critical role of the length of dependency links,” Phys. Rev. Lett. 108, 228702 (2012). 10.1103/PhysRevLett.108.228702 23003664

[28] L. Daqing , K. Kosmidis , A. Bunde , and S. Havlin , “ Dimension of spatially embedded networks,” Nat. Phys. 7, 481–484 (2011). 10.1038/nphys1932

[29] S. M. Pincus , “ Approximate entropy as a measure of system complexity.” Proc. Nat. Acad. Sci. U.S.A. 88, 2297–2301 (1991). 10.1073/pnas.88.6.2297 PMC51218 11607165

[30] H. Dörrie , Triumph der Mathematik: 100 berühmte Probleme aus zwei Jahrtausenden mathematischer Kultur ( Physica-Verlag, 1958).

